# Editorial: Working Dogs: Form and Function

**DOI:** 10.3389/fvets.2019.00351

**Published:** 2019-10-18

**Authors:** Cynthia M. Otto, Mia L. Cobb, Erik Wilsson

**Affiliations:** ^1^Department of Clinical Sciences and Advanced Medicine, University of Pennsylvania, Philadelphia, PA, United States; ^2^Penn Vet Working Dog Center, Philadelphia, PA, United States; ^3^Working Dog Alliance, Melbourne, VIC, Australia; ^4^School of Psychological Sciences, Monash University, Melbourne, VIC, Australia; ^5^Swedish Armed Forces, Stockholm, Stockholm, Sweden

**Keywords:** heatstress, selection, assistance dog, canine athlete, hydration, gastritis, lameness

Dogs have a long history of serving mankind as hunters and protectors. In the last century the unique roles of dogs working in concert with humans has expanded. Dogs now fulfill vital roles to support the concept of One Health, where the health of animals, humans and the environment intersect. These working dogs include assistance dogs that serve to guide and support individuals in their daily lives, detection dogs that use their powerful noses to identify hazards or odors of interest (e.g., explosives, drugs, diseases, and invasive or endangered species) and law enforcement canines that work with police and the military for our safety. In addition to these lines of work, canine athletes compete in professional (e.g., racing) and amateur events. This Research Topic addresses topics relevant to Working Dogs: **Form** and **Function**. As we ask dogs to provide a wide variety of functions, new information to ensure that they are physically and behaviorally equipped for the tasks is vital.

With the need for dogs to work in a wide variety of conditions, emphasis on factors that support optimal performance will enhance the ability of these dogs to have long and healthy careers. In this special issue; Working Dogs: Form and Function, of the 14 papers, eight address key aspects on preservation of physical health (form) and the remaining six highlight topics ranging from olfaction to service dog selection (function).

In military working dogs, heat stroke is one of the most common preventable cause of death (1) or early retirement (2). For working dogs and other canine athletes, heat stress is a well-recognized threat. Dogs generate heat through muscle activity during exercise, often work in environmentally challenging conditions and rely on panting to eliminate heat. Heat stress is a hazard for working dogs and can result in serious injury or death. In this special issue, temperature regulation was the most common topic and also the one that has generated the most interest from the community. Clinically, canine heat stroke is defined by core body temperatures exceeding 41°C (105.8°F) accompanied by central nervous system (CNS) dysfunction (3). Traditionally, rectal temperatures in excess of 105.8°F have been considered consistent with heat stroke, however this data is derived from hospital records rather than field assessments. It is now recognized that dogs actively exercising can reach body temperatures in excess of 105.8°F without any medical consequences or neurologic dysfunction (Robbins et al.; McNicholl et al.). Monitoring temperature can be challenging in the field setting in which dogs are exercising. Rectal temperature is considered the standard for body temperature measurement, however, medical conditions, physiologic states (4) and aversion to thermometer placement can impair the ability to collect this data. Several studies employed the research tool of telemetric core intestinal temperature monitoring (Robbins et al.; Otto et al.). Alternative ways to monitor for increased body temperature include thermography and physiologic signs.

In the study by Zanghi, rectal temperature, non-contact infrared thermography of the eye and contact thermography of the ear were evaluated in 16 Beagles and 16 Labrador retrievers after 30 min of play indoors with controlled temperature of (78–80°F) 25.6–26.7°C. Breed specific variations in temperature were identified at rest and after exercise, with Labradors maintaining higher temperatures and having slower post-exercise temperature recovery than Beagles. Ear and eye temperatures typically underestimated rectal temperature at rest and the relationship between eye temperature and rectal temperature diverged after activity. Play resulted in an average temperature of only 103.8°F (39.9°C), suggesting that the level of exertion was less than typically seen in exercising working dogs (Robbins et al.) but is consistent with dogs doing controlled on leash searches in a shaded environment (Otto et al.). Even with this moderate level of hyperthermia, after 15 min rectal temperatures in the Beagles had returned to baseline, while it required 30 min for Labrador rectal temperatures to return to baseline. Labrador ear and eye temperatures had not reached baseline by 30 min post-exercise.

Although the least invasive measure (eye temperature) does not track rectal temperature after exercise, other clinical signs can be used to identify dogs with hyperthermia and at risk of heat stress. In a field study of exercising working dogs, Robbins et al., evaluated core body temperature (using ingested temperature sensing capsules) and stamina (defined as the ability to withstand high energy demanding activity over extended periods of time). Dogs were exercised for 30 min outdoors in a median temperature of 28.9°C (84°F) and median humidity of 47%. Core body temperature which reached as high as 42.4°C (108.3°F) did not predict stamina, rather respiratory efficiency or ability to eliminate heat without creating profound acid/base disturbances was a major factor influencing stamina. Clinical signs of heat stress included an extended flat (“spade” shaped) tongue, retracted ears, squinty eyes, panting, and shade seeking.

Strategies to reduce heat stress may provide important ways to improve the performance and safety of working dogs that are required to exert themselves under adverse conditions. Two approaches to prevention are to decrease the heat of work and to improve the efficiency of heat exchange. One strategy is to provide more efficient burning fuels through nutritional modification. Current recommendations suggest that protein should represent 24% of metabolizable energy for working dogs. Protein, when fed in excess will be utilized as an energy source. Fats are considered a primary energy source for dogs. Feeding higher fat diets may improve stamina and olfactory ability, but the source of the fat is also important. Saturated fats (i.e., coconut oil) are reported to decrease olfactory acuity, while polyunsaturated fats (i.e., corn oil) improved olfactory efficiency (5, 6). Dietary fat may also impact thermodynamics. Compared to protein, which requires energy, thus generation of heat, to be utilized, fats are metabolized with close to 100% efficiency. The effect of a high fat (57%; corn oil supplemented), low protein diet (18%) on treadmill exercised detection dogs was compared to a high protein, high fat diet (27%:57% ME) and a high protein: low fat (27%:32% ME) (Ober et al.). The dogs fed the low protein, high fat diet maintained a lower core temperature after exercise compared to dogs fed the high protein, low fat diet. Altering dietary components that may help reduce heat generation is one strategy to optimize thermal balance.

A second approach is to maximize heat loss. Dogs eliminate heat through panting; increased salivation and lingual blood flow allow for convection and evaporative cooling (7). As dogs become more dehydrated, salivation is reduced dramatically (8), thus eliminating most of the cooling effect of salivation. Strategies to enhance hydration and prevent dehydration in working dogs have included providing water or flavored electrolyte solution orally or preloading with subcutaneous electrolyte solutions. None of these approaches had been evaluated under field conditions until a cross-over study was conducted with Border Patrol dogs screening vehicles at the Southern Border of the United States (Otto et al.). The Border Patrol dogs that were provided a flavored oral electrolyte solution consumed over twice the volume of liquid on a per weight basis. The dogs that received electrolyte solutions, whether oral or subcutaneous, had indirect markers of improved hydration (higher total CO_2_, and lower packed cell volume and total plasma protein) at the end of the day. No effect of hydration strategy, however, was seen on core temperature.

Environmental conditions play an important role in the development of heat stress in dogs. The conditions during the Border Patrol study were not extreme (median temperature 84.8°F (29.3°C); median humidity 70%). Similarly, the Robbins et al., study evaluated exercise in dogs under median temperature [28.9°C (84°F)] but lower median humidity (47%) and found stamina was influenced by temperature but not humidity. In the Robbins study, core body temperature was not predicted by either temperature or humidity. In a study of racing greyhounds performing anaerobic exercise (running for 15–45 s over 300–731 m), there was an association between environmental temperature and post-race increase in rectal temperature (McNicholl et al.). When the ambient temperature reached 38°C (100.4°F), over one-third (39%) of greyhounds had a rectal temperature >41.5°C (106.7°F). Darker coat color, heavier body weight and male greyhounds were associated with higher post-race rectal temperatures. Humidity was not a predictor of core temperature in dogs in these studies.

Exercise induced hyperthermia represents a spectrum of injury, if severe enough to cause heat stroke, it can be a deadly. Less severe temperature elevations can lead to subtle physiologic changes that accumulate with repeated episodes of exercise induced hyperthermia and can also have serious consequences. In racing sled dogs, gastric dysfunction and development of ulcers (exercise induced gastric disease; EIGD), has been identified as a major cause of death (Davis and Williamson). The mechanism of injury is not proven; however, repeated exercise-induced hyperthermia, stress and excessive gastrin secretion are current hypotheses thought to contribute to altered mucosal permeability and ulcer formation. EIGD is not limited to endurance athletes. In one study, 84% of explosive detection Labradors had evidence of gastric ulcers after 5 consecutive days of exercise (9).

Exercise depends on the coordinated physiology to deliver oxygen to functioning muscles; without an intact musculoskeletal system, the dogs are unable to achieve the physical demands of mobility. Lameness is the asymmetric use of one or more limbs and can result from injury to muscles, bones, joints and nerves. The etiologic diagnosis of lameness has traditionally relied on physical palpation and radiographs, with options for advanced imaging such as magnetic resonance imaging (MRI), computed tomography (CT) and musculoskeletal ultrasound. In working and athletic dogs, subtle lameness can lead to an observable decrement of performance. In a study of guide dogs (Lloyd et al.), musculoskeletal disorders were the most common reason for dogs being withdrawn from service prior to retirement. Frequently physical examination and traditional imaging are not sensitive enough to detect the site of subtle injuries, and whole-body imaging is not economical or practical in most cases. New advances in imaging, include positron emission tomography (PET) imaging and novel applications of traditional computed tomography (CT), may help improve diagnosis and monitoring of musculoskeletal disease or injury. In PET scans, the radiopharmaceutical, fluorine- 18-fluorodeoxyglucose (18F-FDG), is taken up by hypermetabolic cells, allowing identification of regions of disease, inflammation or cellular activity. Traditionally, PET has been used for tracking cancerous cells, studying glucose metabolism or muscle activity. In a report by Mann et al., PET-CT imaging was utilized to pinpoint the source of a subtle lameness in a dog. The injury was then further characterized by musculoskeletal ultrasound. The addition of PET-CT to the diagnostic armamentarium provides novel information on both anatomic location and cellular activity, which is likely to assist with the characterization of elusive and dynamic lameness in canine athletes.

One common cause of mobility disorders in working dogs is lumbosacral (LS) or low back pain. This debilitating condition, like in humans, can be the result of structural abnormalities of the spine or inadequate muscular support in a highly dynamic region subject to repetitive motion during search behavior. Cain et al. compared CT images from working dogs with LS pain (*n* = 11) to those without LS pain (*n* = 5). Using quantitative transverse area ratios of the paraspinal muscles in the LS region, the investigators were able to compare the association of relative muscle mass and presence of muscle asymmetry to the presence of LS pain. Muscle asymmetry was not different between groups, but dogs with LS pain had significantly lower muscle area ratios. The reduction of muscle area could result from pain and disuse but could also present a target for physical rehabilitation. CT screening may be able to identify dogs at risk of injury and allow development of preventative physical conditioning plans.

The **function** of working dogs depends on their career path. Detection dogs rely on efficient and sensitive olfaction to perform their jobs. As reviewed by Angle et al., applications of canine olfaction to the detection of biological volatile organic compounds (VOCs) has been applied to medical diagnosis, therapeutic monitoring, disease outbreak containment, and disease prevention. Endogenous VOCs are generated through metabolic processes of the host and commensal organisms, transported through the blood and released into the environment through various routes including breath, sweat, feces, and urine. The VOC profile can be altered by numerous factors including diet, genetics, environment, and disease states. Dogs are able to detect odors in concentrations as low as one part per trillion, compared to traditional analytic chemistry instruments which are only sensitive in the order of parts per billion. In addition to the recent focus on cancer detection, biomedical detection dogs have been utilized to identify other diseases and pathogens. The approach has been versatile, with dogs working in laboratory environments, hospital environments and in the field. In the laboratory, *in vivo* and *in vitro* samples have been utilized. In this Research Topic, Angle et al. described how dogs could detect the virus that causes bovine viral diarrhea and discriminate it from bovine herpesvirus 1 and bovine parainfluenza virus 3, in cell cultures (*in vitro*). One of the important field applications of canine olfaction is the detection of diseased, invasive or endangered species. In a review of scent detection dogs in conservation settings Beebe et al., described the scope of conservation including detection of live wildlife, carcass detection for birds, and bats around wind turbines, detection of scats, pathogens, and other biological materials, and detecting the presence/absence, and relative abundance, of plants and wildlife.

A hurdle faced by all disciplines of working dogs is the ability to identify the best dog for the job. It is not unusual to screen hundreds of shelter dogs to identify those with the physical and behavioral traits necessary for success (10). Breeding programs offer some advantages through the ability to select for heritable health and behavioral traits and control the early developmental period of potential working dogs. There is a movement to develop a National Breeding Cooperative in the United States to provide a larger pool of available working dogs to meet the growing demands (11). Selection criteria are important for both breeding programs and for dogs selected from shelters. In the review by Beebe et al., selection parameters identified for conservation detection dogs share several similarities with other types of detection and some assistance dogs. The categories for selection include biological traits, psychological processes and social contexts. Most detection dogs and traditional assistance dogs are medium to large in stature, however, in the US, as the number of psychiatric dogs has increased, the number of small breed assistance dogs has increased dramatically (Walther et al.). Detection dogs (e.g. conservation, search and rescue, explosives, narcotics etc.) are often selected from working lines with strong olfactory capability and an unflagging desire to work for toys or in some cases, food. Normal hearing is necessary for optimal communication between dogs and handlers in most working careers. Assistance dogs rely more on visual cues than detection dogs, but good vision is essential for all working dogs. Conservation and detection dogs are often selected based on the psychological dimensions of persistence (the ability to sustain working motivation) and boldness (the response to unfamiliar stimuli and environments); although validated quantitative measures of these traits are generally lacking. A standardized, validated, and widely used survey instrument that measures the frequency and/or severity of most common behavior problems in dogs has been used to characterize assistance (Serpell and Duffy) and search dogs (12). In a study by Serpell and Duffy, predictors of behavioral traits in Guide Dogs being raised in volunteer's homes were retrospectively evaluated; German shepherd dogs had an increase in aggression toward strangers, but not familiar individuals between 6 and 12 months. Traumatic events during the 6–12 month stage were associated with more fear in the dogs. Being raised in a multidog household was associated with more positive behavioral traits.

The third aspect (social characteristics) of selection of conservation dogs, which is likely applicable to all working dog teams, focuses on correctly pairing dog-handler teams based on the experience and psychological traits of both the dog and the handler (Beebe et al.). In addition to their role as a mobility aid, guide dogs provide also provide other benefits that influence the success of the match. A successful match of guide dog and handler relies on the mobility and orientation teamwork, social interactions with the handler, other people and animals, and adaptation to the home environment. Over one quarter of guide dogs were identified as mismatched with handlers. Generally, the mismatch was apparent within 10 months of the match. The most common behavioral reason for return of a dog was poor home/social behavior. As with the importance of the role of the handler, the puppy raiser's experience also has an important impact on positive behavioral traits of the dog; the more experienced the puppy raiser, the more likely the dog will exhibit positive behavioral traits (Serpell and Duffy).

Preparing dogs and handlers for any type of working career is challenging. There is an increasing demand for both assistance and detection dogs. In response to this demand, many new organizations have cropped up, particularly in the field of assistance dogs (Walther et al.). In the United States, there are no published national standards for assistance dogs. Although the international organizations, Assistance Dogs International (ADI; https://assistancedogsinternational.org/) and International Guide Dog Federation (IGDF; https://www.igdf.org.uk/), have established basic guidelines and an accreditation process, these are voluntary. Many of the smaller organizations are not accredited and appear to have a high turnover (Walther et al.). There is a global shortage of detection dogs that is attributed to increased international demand (11). The National Institute of Standards and Technology (NIST; https://www.nist.gov/topics/forensic-science/dogs-sensors-subcommittee) has been working to convert Scientific Working Group on Dogs and Orthogonal detector Guidelines (SWGDOG; http://swgdog.fiu.edu/) into standards for detection dogs to ensure that the dogs that are serving in these important roles meet defined performance and care standards. In order to create standards, continued research into the form and function of these dogs is vital. These 14 studies published in this Research Topic have added to our knowledge to support the advancement of working dog science, but as all studies do, they raise more questions that we need to address. We are proud to be able to promote the science and dissemination of knowledge in the Frontiers open source venue and invite you to contribute your science to the next Working Dog Research Topic.

## Author Contributions

This editorial was authored by CO with review and input by MC and EW.

### Conflict of Interest

The authors declare that the research was conducted in the absence of any commercial or financial relationships that could be construed as a potential conflict of interest.
