# Corrigendum: Editorial: Working Dogs: Form and Function

**DOI:** 10.3389/fvets.2020.00093

**Published:** 2020-02-26

**Authors:** Cynthia M. Otto, Mia L. Cobb, Erik Wilsson

**Affiliations:** ^1^Department of Clinical Sciences and Advanced Medicine, University of Pennsylvania, Philadelphia, PA, United States; ^2^Penn Vet Working Dog Center, Philadelphia, PA, United States; ^3^Working Dog Alliance, Melbourne, VIC, Australia; ^4^School of Psychological Sciences, Monash University, Melbourne, VIC, Australia; ^5^Swedish Armed Forces, Stockholm, Stockholm, Sweden

**Keywords:** heatstress, selection, assistance dog, canine athlete, hydration, gastritis, lameness

In the original article, McNicholl et al. was incorrectly mentioned in the **6th paragraph**. The correct article should be Ober et al. The fully corrected paragraph appears below.

“Strategies to reduce heat stress may provide important ways to improve the performance and safety of working dogs that are required to exert themselves under adverse conditions. Two approaches to prevention are to decrease the heat of work and to improve the efficiency of heat exchange. One strategy is to provide more efficient burning fuels through nutritional modification. Current recommendations suggest that protein should represent 24% of metabolizable energy for working dogs. Protein, when fed in excess will be utilized as an energy source. Fats are considered a primary energy source for dogs. Feeding higher fat diets may improve stamina and olfactory ability, but the source of the fat is also important. Saturated fats (i.e., coconut oil) are reported to decrease olfactory acuity, while polyunsaturated fats (i.e., corn oil) improved olfactory efficiency (5, 6). Dietary fat may also impact thermodynamics. Compared to protein, which requires energy, thus generation of heat, to be utilized, fats are metabolized with close to 100% efficiency. The effect of a high fat (57%; corn oil supplemented), low protein diet (18%) on treadmill exercised detection dogs was compared to a high protein, high fat diet (27%:57% ME) and a high protein: low fat (27%:32% ME) (Ober et al.). The dogs fed the low protein, high fat diet maintained a lower core temperature after exercise compared to dogs fed the high protein, low fat diet. Altering dietary components that may help reduce heat generation is one strategy to optimize thermal balance.”

The authors apologize for this error and state that this does not change the scientific conclusions of the article in any way. The original article has been updated.

